# Effects of Tamoxifen vs. Toremifene on fatty liver development and lipid profiles in breast Cancer

**DOI:** 10.1186/s12885-021-08538-5

**Published:** 2021-07-10

**Authors:** Dandan Song, Yingying Hu, Biyu Diao, Rongrong Miao, Baodan Zhang, Yangjun Cai, Hanqian Zeng, Yuru Zhang, Xiaoqu Hu

**Affiliations:** 1 Department of Breast Surgery, The First Affiliated Hospital of Wenzhou Medical University, Wenzhou, Zhejiang, China; 2 Department of Oncology Surgey, Wenzhou People’s Hospital, Wenzhou, Zhejiang, China; 3 Department of Ultrasound Imaging, The First Affiliated Hospital of Wenzhou Medical University, Wenzhou, Zhejiang, China; 4 Department of Urinary Surgery, The First Affiliated Hospital of Wenzhou Medical University, Wenzhou, Zhejiang, China; 5 Department of Thyroid and Breast Surgery, Taizhou Hospital of Zhejiang Province, Taizhou, Zhejiang, China

**Keywords:** Tamoxifen, Toremifene, Fatty liver, Lipid profiles

## Abstract

**Background:**

Tamoxifen (TAM) and Toremifene (TOR), two kinds of selective estrogen receptor modulators (SERMs), have equal efficacy in breast cancer patients. However, TAM has been proved to affect serum lipid profiles and cause fatty liver disease. The study aimed to compare the effects of TAM and TOR on fatty liver development and lipid profiles.

**Methods:**

This study performed a retrospective analysis of 308 SERMs-treated early breast cancer patients who were matched 1:1 based on propensity scores. The follow-up period was 3 years. The primary outcomes were fatty liver detected by ultrasonography or computed tomography (CT), variation in fibrosis indexes, and serum lipid profiles change.

**Results:**

The cumulative incidence rate of new-onset fatty liver was higher in the TAM group than in the TOR group (113.2 vs. 67.2 per 1000 person-years, *p* < 0.001), and more severe fatty livers occurred in the TAM group (25.5 vs. 7.5 per 1000 person-years, *p* = 0.003). According to the Kaplan-Meier curves, TAM significantly increased the risk of new-onset fatty liver (25.97% vs. 17.53%, *p* = 0.0243) and the severe fatty liver (5.84% vs. 1.95%, *p* = 0.0429). TOR decreased the risk of new-onset fatty liver by 45% (hazard ratio = 0.55, *p* = 0.020) and showed lower fibrotic burden, independent of obesity, lipid, and liver enzyme levels. TOR increased triglycerides less than TAM, and TOR increased high-density lipoprotein cholesterol, while TAM did the opposite. No significant differences in total cholesterol and low-density lipoprotein cholesterol are observed between the two groups.

**Conclusions:**

TAM treatment is significantly associated with more severe fatty liver disease and liver fibrosis, while TOR is associated with an overall improvement in lipid profiles, which supports continuous monitoring of liver imaging and serum lipid levels during SERM treatment.

## Background

In China, the breast cancer morbidity rate has increased rapidly and has become the most prevalent cancer among women in recent years. For Chinese women, the average age at diagnosis is 40 to 50 years, more than 10 years younger than the age reported in western countries, and premenopausal cases account for most breast cancer patients [1, 2]. Selective estrogen receptor modulators (SERMs), including tamoxifen (TAM) and toremifene (TOR), have been verified to have similar efficacy for premenopausal and postmenopausal estrogen receptor-positive breast cancer patients [3, 4]. Since endocrine therapy is currently recommended for 5–10 years, the side effects of long-term use of TAM and TOR need to be acknowledged. These include the risk of endometrial cancer, venous thrombosis, fatty liver disease, lipid dysfunction, and interference with infant development and breastfeed [5–7]. Several previous studies have shown that during 3–5 years of follow-up, 30.4–52.6% of patients undergoing TAM treatment developed fatty liver [8–11]. Some researchers have explored the effect of TAM on serum lipid profiles; however, their findings are not consistent. Overall, TAM increases serum triglyceride levels and low-density lipoprotein cholesterol, which are important risk factors for cardiovascular events [12]. Although the pathological mechanism is not yet precise, TAM is thought to affect lipid metabolism by promoting triglyceride synthesis and aggregation, reducing fatty acid oxidation, and inhibiting estrogen synthesis [13–15]. TOR, a chlorinated derivative of TAM, does not increase intracellular concentrations of triglyceride as TAM does in vitro [16]. However, data on the effects of TAM and TOR on lipid abnormality were inconsistent, and the investigations reported so far were performed in Western or postmenopausal women. To date, there are no data regarding the comparisons of fatty liver and serum lipids abnormality caused by TOR and TAM with a large sample size in premenopausal breast cancer. Herein, this retrospective propensity score-matched cohort study was performed to compare the effect of TAM and TOR on the risk of newly developed fatty liver and the change of serum lipid profiles.

## Methods

### Patient cohort

**Fig. 1:**
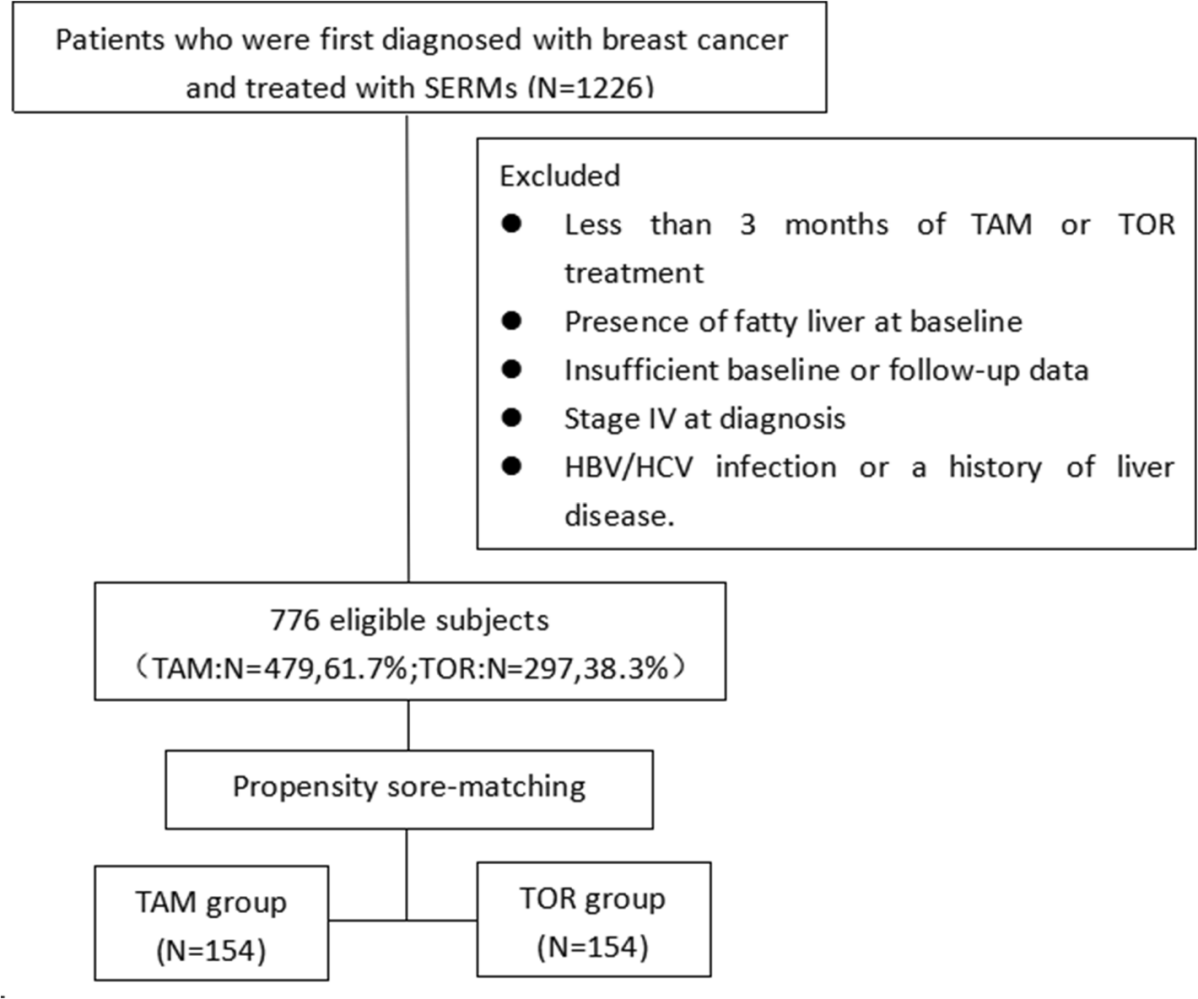
Patient selection algorithm

A propensity score-matched (PSM) study was performed by reviewing the electronic medical records of a single university-affiliated, tertiary-level institution (Fig. 1). From January 2011 to June 2017, a total of 1226 adult women underwent surgery, were diagnosed with breast cancer, and then received TAM (20 mg/day) or TOR (60 mg/day) as adjuvant endocrine therapy. The index date of entry into the study was defined as the date of the first prescription for TAM or TOR. The baseline data of the subjects, from before surgery, were retrieved, including demographic, smoking status, alcohol consumption, complications, pathology reports, treatment regimens, and liver ultrasonography(USG) or computed tomography (CT), laboratory values. Patients who met any of the following criteria were excluded: less than 3 months of TAM or TOR treatment; the presence of fatty liver at baseline, defined by USG or CT; insufficient baseline or follow-up information; hepatitis B surface antigen or anti-hepatitis C virus antibody positivity; the history of liver disease; a prescription for any drug that affects lipid or liver enzyme levels; Stage IV status at diagnosis; smoker; or significant alcohol intake (> 20 g/day). The above patients would be followed-up for 3 years, or until SERM therapy was discontinued. Seven hundred seventy-six patients met the inclusion criteria (TAM group *N* = 479; TOR group *N* = 297), and 154 patients were eventually included in each group after PSM (Fig. 1).

### Measurements

All individuals underwent liver USG or CT at baseline and at least once at the annual follow-up visit, with both being performed by experienced sonographers and radiologists who were blinded to the study. USG was used to diagnose fatty liver based on the increased hepatic echogenicity compared to the cortex of the right kidney and the echo loss of the intrahepatic vessel walls. Fatty liver measured by CT is divided into the following levels: (1) mild-to-moderate: CT ratio of liver to the spleen is less than 0.9 and (2) severe: CT ratio less than 0.5 or the liver parenchyma appearing darker than the hepatic vessels [17, 18]. The primary endpoint of this study was the new identification of the fatty liver. Fatty liver graded as severe by CT was defined as the secondary endpoint.

Liver biopsy is the gold standard to determine the degree of liver fibrosis. Compared with the risks and costs associated with liver biopsy, noninvasive diagnoses provide a safe and practical approach to quantify liver fibrosis. Two simple, noninvasive indexes were used to assess liver fibrosis—aspartate aminotransferase-platelet ratio index (APRI) and Fibrosis 4 index (FIB-4), which are proven to provide satisfactory diagnostic performance for detecting liver fibrosis [19, 20]. There follows the formulas: APRI = [(AST/upper limit of normal)/platelet count(109/L)] × 100; FIB-4 = [age (years) × AST (U/L)]/ [platelet count (109/L) × ALT1/2 (U/L)]. These indexes include liver enzymes and platelet counts as parameters, which can be interpreted as liver fibrosis leading to liver dysfunction and portal hypertension leading to platelet accumulation in the spleen [21]. Two risk grades (low, medium, or high) were set up for each score according to the cut-off values described in the original publication. The cut-off values are 0.5 for the APRI and 1.30 for the FIB-4 [22, 23].

Laboratory tests were performed at baseline and during routine annual visits, and data from individuals were collected at the end of SERM treatment.

### Statistical analysis

Continuous data with a normal distribution are described as the mean ± standard deviation (SD), and a paired t-test was applied to compare the differences between the two groups. Otherwise, the median [interquartile range] and Wilcoxon rank-sum test were employed. Categorical data are presented as the number of cases (%), and the McNemar-Bowker test was utilized to compare the differences between the two groups. Propensity score matching was performed by using logistic regression. Based on the previous literature and clinical knowledge, potential risk factors leading to the occurrence of fatty liver were included as propensity score covariates, as follows: age, body mass index (BMI), ALT/AST ratio, and high-density lipoprotein-cholesterol (HDL-C) concentration. Kaplan-Meier curves were plotted to compare the development of fatty liver between the matched groups. Univariable and multivariable Cox proportional hazards regression models were utilized to identify independent factors related to the outcome variables. The changes in serum fibrosis markers were analyzed by linear mixed-effects regression models with an unstructured covariance pattern. The models included the treatment (TAM or TOR), time as fixed effects; age, BMI, diabetes, hypertension and ALT/AST ratio as fixed covariables; study subjects as a random effect. The changes in the serum lipid profiles over time were compared between the TAM and TOR groups by repeated-measures analysis of variance. A two-sided *P*-value of < 0.05 was considered significant. Statistical analyses were performed using SAS 9.4 (SAS Institute Inc., Cary, North Carolina, USA). The power of test was performed using R-Studio (1.2.5001), and the result was about 30% (Alpha = 0.05).

## Result

### Baseline characteristics after propensity score matching analysis

The baseline characteristics of 308 participants in the score-matched cohort are shown in Table 1, and the main laboratory values did not differ between the two groups. There were no significant differences in mean age, BMI, menstrual status, the prevalence of hypertension or diabetes between the TAM and TOR groups. The breast cancer at diagnosis was mainly staged 1 or 2 and estrogen-receptor-positive in both groups. However, the TOR group included more estrogen-receptor-positive subjects (94.2% vs 98.7%, *p* = 0.032), while the TAM group included more advanced breast cancer subjects (*p* = 0.024) and more subjects who received chemotherapy (82.5% vs 72.1%, *p* = 0.030). The median duration of SERM therapy was 36 months. Liver fibrosis indexes and medium or high fibrosis proportions showed no significant differences in the two groups. (Table 2).

**Table 1:** Baseline characteristics and treatment in the PSM cohorts

Variables	Tamoxifen (***n*** = 154)	Toremifene (***n*** = 154)	***P***-value
Age, years	44.0 [40.0–48.0]	44.0 [40.0–48.0]	0.890
BMI, kg/m^2^	22.1 [20.6–23.7]	21.7 [20.4–23.5]	0.444
Menstrual status			0.239
Premenopausal	146 (94.8)	150 (97.4)	
Postmenopausal	8 (5.2)	4 (2.6)	
Hypertension	14 (9.1)	14 (9.1)	1.000
Diabetes	0 (0.0)	3 (1.9)	0.246
Cancer stage			0.024
0^a^	23 (14.9)	22 (14.3)	
1	48 (31.2)	72 (46.8)	
2	53 (34.4)	43 (27.9)	
3	30 (19.5)	17 (11.0)	
Pathologic type			0.339
Ductal or lobular carcinoma in situ	23 (14.9)	26 (16.9)	
Invasive ductal or lobular carcinoma	123 (79.9)	114 (74.0)	
Others^b^	8 (5.2)	14 (9.1)	
Hormone receptor status			
ER positive	145 (94.2)	152 (98.7)	0.032
PR positive	132 (85.7)	140 (90.9)	0.156
HER-2 positive	26 (16.9)	28 (18.2)	0.764
Therapeutic schedule			
Chemotherapy	127 (82.5)	111 (72.1)	0.030
Radiation therapy	53 (34.4)	67 (43.5)	0.102
Trastuzumab	9 (5.8)	14 (9.1)	0.278
Ovarian function suppression	16 (10.4)	27 (17.5)	0.071
Traditional Chinese medicine	29 (18.8)	36 (23.4)	0.328
Endocrine therapy duration, months	36.0 [18.2–36.0]	36.0 [28.1–36.0]	< 0.001
Laboratory values			
Total cholesterol, mmol/L	4.8 [4.2–5.5]	4.9 [4.5–5.4]	0.483
Triglyceride, mmol/L	1.0 [0.7–1.6]	0.9 [0.7–1.2]	0.055
HDL-C, mmol/L	1.4 [1.2–1.7]	1.4 [1.3–1.7]	0.245
LDL-C, mmol/L	2.7 [2.2–3.3]	2.8 [2.4–3.2]	0.913
ALT, IU/L	13.0 [10.0–17.0]	13.0 [10.0–16.0]	0.656
AST, IU/L	18.0 [16.0–21.0]	18.0 [16.0–21.0]	0.698
ALT/AST ratio	0.7 [0.6–0.9]	0.7 [0.6–0.8]	0.892
Fasting glucose, mmol/L	5.3 [4.9–5.7]	5.3 [4.8–5.8]	0.851

Data are presented as the mean ± standard deviation, median [interquartile range] or number (%)

Abbreviations: *BMI* body mass index; *ER* estrogen receptor; *PR* progesterone receptor; *HER-2* Human Epidermal Growth Factor Receptor 2; *HDL-C* high-density lipoprotein-cholesterol; *LDL-C* low-density lipoprotein-cholesterol; *ALT* alanine aminotransferase; *AST* aspartate aminotransferase;

^a^Ductal or lobular carcinoma in situ and Paget’s disease were included in stage 0.

^b^Other pathological types included mucinous, tubular, papillary, and Paget’s disease.

**Table 2:** The fibrosis indexes of the study subjects in the PSM cohorts

Variables	Tamoxifen (***n*** = 154)	Toremifene (***n*** = 154)	***P***-value
APRI at baseline	0.23 [0.19–0.30]	0.22 [0.18–0.27]	0.052
APRI at follow-up time	0.30 [0.26–0.37]	0.25 [0.22–0.34]	*< 0.001*
*P*-value	*< 0.001*	*< 0.001*	
Medium or high APRI at baseline	4 (2.6)	3 (1.9)	1.000
Medium or high APRI at follow-up time	15 (9.7)	4 (2.6)	*0.009*
*P*-value	*0.009*	1.000	
FIB-4 at baseline	0.99 [0.79–1.20]	0.93 [0.77–1.11]	0.095
FIB-4 at follow-up time	1.13 [0.92,1.35]	1.04 [0.87,1.30]	*0.034*
*P*-value	*< 0.001*	*< 0.001*	
Medium or high FIB-4 at baseline	28 (18.2)	18 (11.7)	0.110
Medium or high FIB-4 at follow-up time	45 (29.2)	37 (24.0)	0.302
*P*-value	*0.023*	*0.004*	

Statistically significant values are highlighted in italics

Data are presented as the mean ± standard deviation, median [interquartile range] or number (%)

Abbreviations: *APRI* aspartate aminotransferase-platelet ratio index; *FIB-4* Fibrosis- 4 index

### Analysis of fatty liver development

**Fig. 2:**
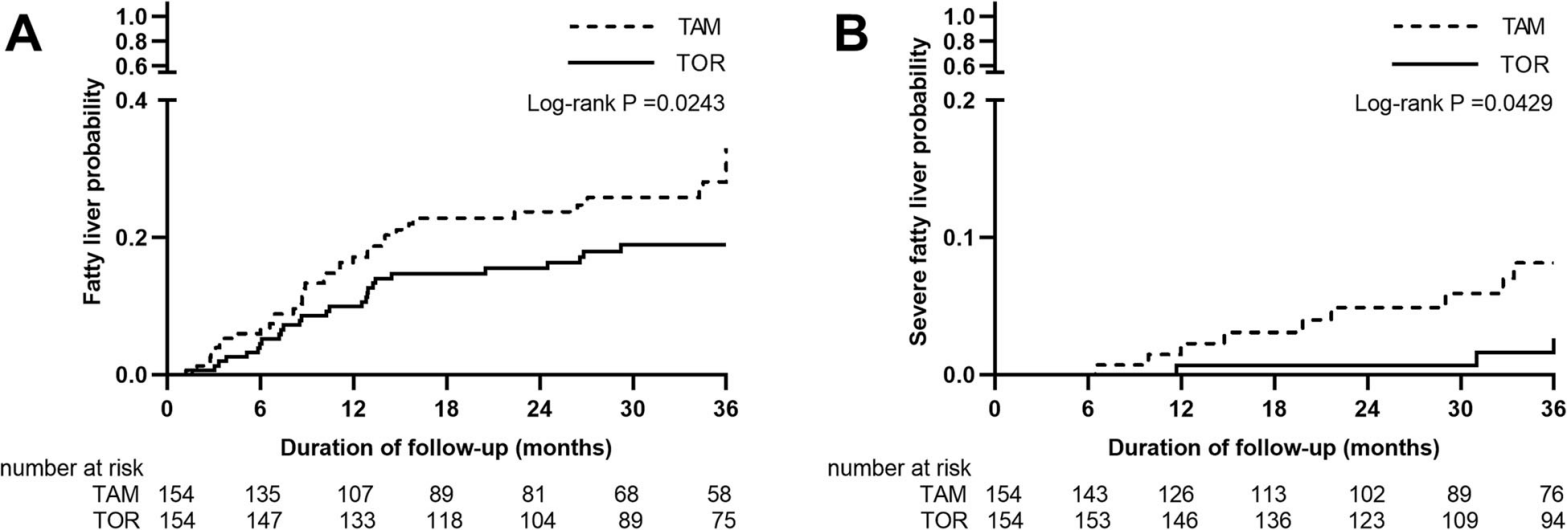
In propensity score-matched pairs, Kaplan-Meier curves for the probability of (**A**) any level of new-onset fatty liver diagnosed by USG or CT and (**B**) severe fatty liver

In the propensity score-matched cohorts, 40 cases in the TAM group and 27 cases in the TOR group developed fatty liver. Over the total 755.3 person-years in the matched cohort, the cumulative incidence rate of new-onset fatty liver was higher in the TAM group than in the TOR group (113.2 vs. 67.2 per 1000 person-years, *p* < 0.001). Most of the severe fatty livers occurred in the TAM group (25.5 vs. 7.5 per 1000 person-years, *p* = 0.003). According to the Kaplan-Meier curves, compared with the TOR group, the TAM group had more rapidly increasing probabilities of any level of fatty liver, particularly within the first 16 months of treatment (25.97% vs. 17.53%, log-rank *p* = 0.0243, Fig. 2A). A significant increase in the probability of severe fatty liver was detected in the TAM group compared with the TOR group (5.84% vs. 1.95%, log-rank *p* = 0.0429, Fig. 2B).

Variations in liver fibrosis indicators are shown in Table 2. At the end of SERM treatment, APRI and FIB-4 were elevated from baseline in both groups (*p* < 0.001). Serum fibrosis markers at baseline showed no significant difference between the two groups, whereas, at the end of follow-up, APRI and FIB-4 in the TAM group were higher than those in the TOR group with statistical differences (APRI, 0.30 vs. 0.25, *p* < 0.001; FIB-4, 1.13 vs. 1.04, *p* = 0.034). As for the proportion of medium or high fibrosis statistically increased in the TAM group measured by APRI and FIB-4, while increased in the TOR group measured by FIB-4. The patients who received TAM had a higher proportion of medium or high fibrosis than those who received TOR, as measured by the APRI (9.7% vs. 2.6%, *p* = 0.009). However, when comparing FIB-4, there was no significant difference in the degree of liver fibrosis between the two groups (29.2% vs. 24.0%, *p* = 0.302). In linear mixed-effects regression models, the effect of TAM on the increase in fibrosis indexes was more obvious than that of TOR (APRI *p* = 0.034; FIB-4 *p* = 0.005). Besides, the trend of the APRI over time varied depending on the different SERM treatments (*p* = 0.042).

The univariate Cox proportional hazards models showed that TOR use (versus TAM) (hazard ratio [HR] = 0.58, *p* = 0.026) significantly reduced the risk of new-onset fatty liver. In contrast, age, BMI, the ALT/AST ratio, triglyceride (TG), and diabetes increased the risk of fatty liver development. In multivariate analyses, compared with TAM use, TOR use was also associated with a decreased risk of newly developed fatty liver (HR = 0.55, *p* = 0.020), and the ALT/AST ratio, BMI also remained significant independent predictors (Table 3).

**Table 3:** Independent predictors associated with new-onset fatty liver in the PSM cohorts

Variables	Univariate		Multivariate	
	HR (95% CI)	*P*-value	HR (95% CI)	*P*-value
TOR use (versus TAM)	0.58 (0.35–0.94)	*0.026*	0.55 (0.33,0.91)	*0.020*
Age	1.05 (1.01–1.09)	*0.008*	1.03 (0.98,1.07)	0.248
BMI	1.26 (1.16–1.37)	*<.0001*	1.23 (1.13,1.35)	*<.0001*
ALT/AST ratio	4.74 (1.59–14.11)	*0.005*	3.93 (1.16,13.29)	*0.028*
HDL-cholesterol	0.52 (0.25–1.08)	0.079		
Triglyceride	1.36 (1.06–1.75)	*0.016*	1.27 (0.94,1.72)	0.126
Endocrine therapy duration	0.99 (0.96–1.02)	0.649		
Radiotherapy	0.65 (0.39–1.09)	0.106		
Chemotherapy	1.18 (0.66–2.13)	0.575		
Diabetes	4.35 (1.06–17.88)	*0.041*	4.16 (0.87,19.95)	0.075
Hypertension	1.56 (0.74–3.26)	0.241	0.97 (0.42,2.25)	0.952
Cancer stage (versus stage 0)				
1	0.60 (0.26–1.37)	0.225		
2	2.05 (0.98–4.26)	0.055		
3	0.94 (0.36,2.45)	0.906		

Statistically significant values are highlighted in italics

Abbreviations: *HR* hazard ratio; *CI* confidence interval; *TAM* tamoxifen; *TOR* toremifene; *BMI* body mass index; *ALT* alanine aminotransferase; *AST* aspartate aminotransferase; *HDL-C* high-density lipoprotein-cholesterol

### Changes in lipid profiles

**Fig. 3:**
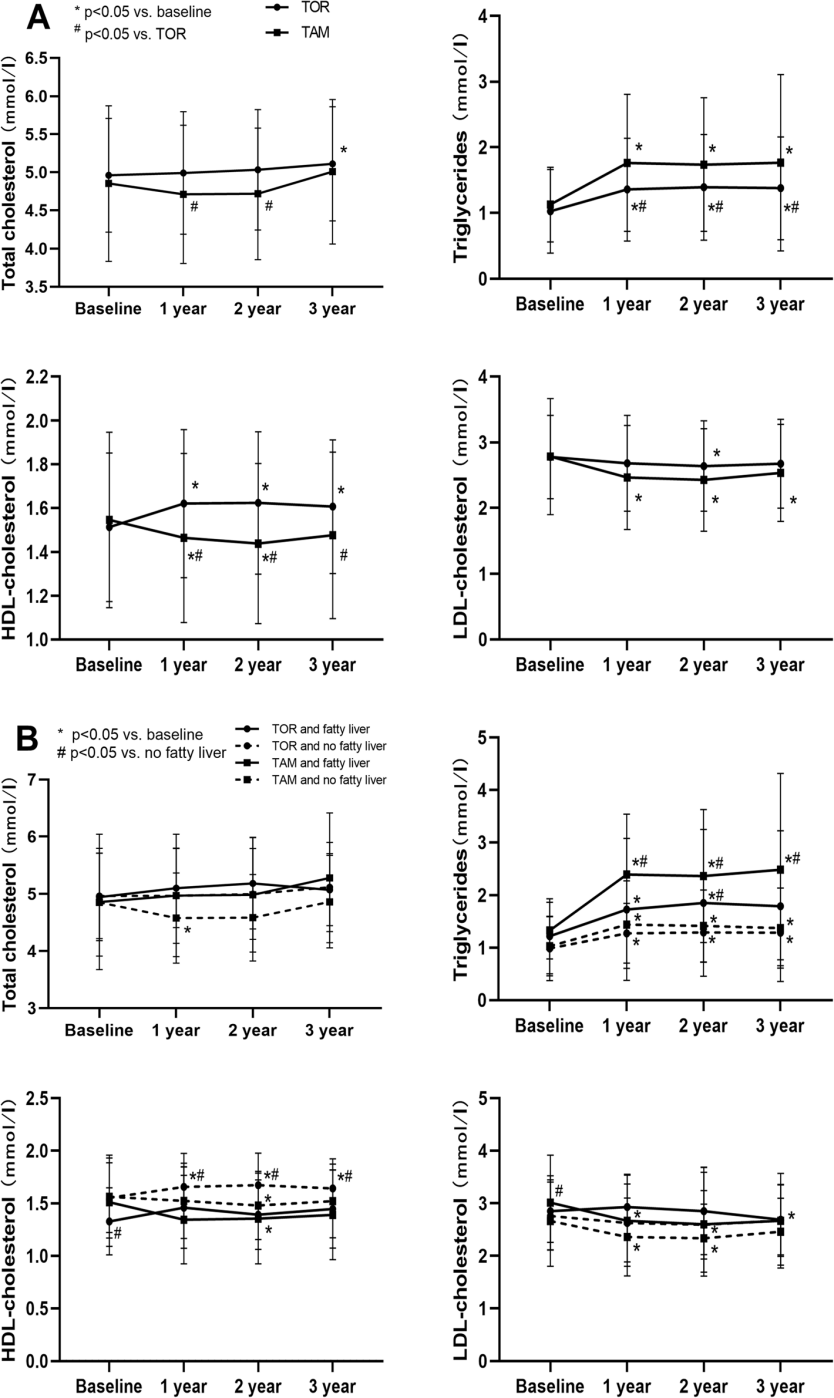
Serum lipid profiles changes (**A**) grouped by treatment (TAM or TOR) and (**B**) grouped by treatment and fatty liver

We analyzed the lipid profile data of subjects who had completed 3 years of follow-up (TAM group *N* = 59; TOR group *N* = 88). Longitudinal changes after administration are shown in Fig. 3A. There was a notable increase in TG levels during the first year in both groups that remained unchanged thereafter compared with baseline. Besides, at any point after 1 year of treatment, the TG levels in the TAM group were significantly higher than those in the TOR group. Similarly, the HDL-C in the TOM group increased while in the TAM group decreased in the first year and then were maintained, with a significant difference between the two groups. Low-density lipoprotein-cholesterol (LDL-C) levels in the two groups significantly decreased and were lower in the TAM group, but there was no significant difference between the two groups. Compared with baseline, TAM and TOR had no noticeable effect on total cholesterol (TC), but the TC of the TAM group was significantly lower than that of the TOR group in the second and third years. When the changes in lipids profiles were analyzed in groups according to fatty liver, the patterns of the changes were similar between participants with new-onset fatty liver and those without in both groups (Fig. 3B). TG levels increased in both groups in individuals with fatty liver, and the TG levels in the TAM group were higher than those in the TOR group regardless of whether the subjects had fatty liver. Regardless of fatty liver, HDL-C of the TOR group was increased while that of the TAM group was decreased.

## Discussion

In this retrospective study, we found that compared with TOR use, TAM use significantly increased the risk of new-onset fatty liver, as well as the risk of severe fatty liver, during a 3-year follow-up. The adverse consequence of TAM treatment was independent of other risk predictors for fatty liver, including age, BMI, TG level and diabetes. Meanwhile, the increase in noninvasive liver fibrosis scores showed statistically significant differences between the two groups. Regarding lipid changes, TAM significantly decreased LDL-C levels, both TAM and TOR significantly increased TG levels, and TOR increased HDL-C while TAM did the opposite. When TG levels and HDL-C were analyzed in groups according to fatty liver, the patterns of the changes were similar.

The incidence of fatty liver in subjects treated with TAM ranged from 30.4 to 52.6% during the 3–5 years of follow-up, and fatty liver was detected within the first 2 years in most cases [8–11]. However, the effect of TOR on fatty liver disease is unclear; a Japanese study suggested a 7.7% incidence of TOR-induced fatty liver based on CT [24]. The results of this research showed that the incidence of TAM treatment-related fatty liver was 25.97%, lower than previous findings, which may be attributed to the fact that most of subjects were younger premenopausal women. In contrast, the incidence of TOR treatment-related fatty liver was was 17.53%, which may be due to the higher sensitivity of USG combined with CT in the diagnosis. Moreover, similar to previous studies, the risk of new-onset fatty liver in both groups increased sharply within the first 1.5–2 years. Few studies have compared the risk of fatty liver disease between TAM and TOR. In this research, TAM was an independent factor for increased risk of fatty liver disease compared with TOR. In contrast, prospective studies by Yang et al.(41.3% vs 50.2%, *p* = 0.45), and Jin et al.(31.9% vs 26.7%, *p* = 0.581), reported that no increased risk of TAM than TOR [25, 26]. However, the primary endpoint of both two studies was not the development of fatty liver disease, and patients with fatty liver disease were not excluded at baseline; meanwhile, more postmenopausal, older, high-BMI patients were enrolled in the TOR group in Yang et al.’s study. The results of this study were reliable because of the better consistency of baseline data after PSM and the higher sensitivity of the combined diagnosis of USG and CT.

NAFLD includes liver diseases ranging from simple steatosis to advanced fibrosis, cirrhosis, and eventually hepatic carcinoma. The “two-hit” hypothesis of NAFLD is widely accepted as the pathogenesis. Previous studies have shown that estrogen has a protective effect against NAFLD development, and TAM increases hepatic fat content (first hit) by blocking the role of estrogen in lipid homeostasis [27, 28]. Obesity, insulin resistance also play a role in the first hit of NAFLD, and TAM may also contribute to hepatic steatosis by increasing serum TG levels. In this context, as a secondary agent, TAM induces inflammation, fibrosis, or necrosis for NAFLD to develop [27]. An in vitro experiment showed that TOR did not increase intracellular triglyceride levels as much as TAM [15]. This study showed that TAM use (versus TOR use), BMI, serum TG levels and diabetes were associated with the occurrence of fatty liver, which was consistent with the pathological mechanism of NAFLD. Although TG levels and diabetes were not significant predictors in the multivariate analyses, it cannot be excluded that the failure to show statistical significance might be due to the insufficient sample size.

It has been reported that advanced fibrosis is a crucial prognostic factor for NAFLD. Several studies have revealed that APRI and FIB-4 can stratify the risks of liver-related morbidity and mortality [29]. This study found that liver fibrosis scores showed statistically significant differences between the TAM and TOR groups, independent of obesity and diabetes. Measured by the APRI, the proportion of significant fibrosis was significantly greater in those who underwent TAM treatment. Previous studies have shown that APRI and FIB-4 are more advantageous in excluding advanced fibrosis in NAFLD patients, and the positive predictive value of APRI is higher than that of FIB-4, which may explain why the proportion of significant fibrosis was significant with APRI but not with FIB-4 [30, 31]. Considering NAFLD could develop into nonalcoholic steatohepatitis or even irreversible cirrhosis, the results of this research probably support the monitoring of fatty liver development during SERM treatment. Since TOR showed a favorable pattern versus TAM in NAFLD progression, we suggest TOR as adjuvant endocrine therapy for premenopausal estrogen-receptor-positive breast cancer patients, especially those with obesity, abnormal TG levels and insulin resistance. Up to now, the correlation between SERM-associated NAFLD and breast cancer prognosis has remained contentious [32, 33]. In a meta-analysis, endocrine treatment(including SERM and aromatase inhibitor) associated with NAFLD showed no significant impact on disease-free survival (DFS) and overall survival(OS). In contrast, non-endocrine treatment associated with NAFLD had a significant correlation with poor OS [34]. Although NAFLD was considered to increase the risk of cancer, its negative impact on survival may be partly counteracted by its protective effect on liver metastases [35]. In patients with NAFLD, insulin-like growth factor-1(IGF-1) levels are low, reducing anti-estrogen resistance resulting from activation of Akt and mitogen-activated protein kinase (MAPK) signaling networks [34].

Previous literature has reported adverse effects of SERMs on lipid profiles, but the outcomes have not been consistent. TAM typically induces a decrease in TC and LDL-C levels and an increase in TG level, whereas HDL-C concentration has been reported to be increased, decreased, or unchanged [12]. Similar to the results of tamoxifen studies, TOR usually reduces TC and LDL-C levels while increases TG and HDL-C cholesterol [36, 37]. In this study, the effects of TAM and TOR on the trends of lipids were consistent with the above rules. In addition, TOR increased triglycerides less than TAM, and TOR increased HDL-C while TAM did the opposite; these trends were maintained throughout the treatment period. Other outcomes of a previous study confirmed that TOR has better effects on lipid profiles than TAM, especially on TG and HDL-C levels [36–38]. A Japanese crossover experiment powerfully illustrated that the lipid profile changes associated with TOR are better than those associated with TAM. After a year of SERMS treatment, compared with the TAM group(*N* = 121), HDL-C was significantly higher, and TG was significantly lower in the TOR group(*N* = 76). After a year of the crossover, TG levels decreased while HDL-C levels increased in subjects switched from TAM to TOR(*N* = 57); in contrast, TG levels increased in subjects switched from TOR to TAM(*N* = 23) [39, 40].

HDL-C is commonly considered good cholesterol due to its improvement of atherosclerotic vascular lesions and the promotion of reverse cholesterol transport [41]. In epidemiological studies, elevated serum levels of HDL-C are associated with a decreased CVD risk and its sequelae [41]. Conversely, a high LDL-C level is a critical risk factor relative to CVD [42]. This study proved that TOR improves HDL-C levels while not significantly increasing TC and decreasing LDL-C levels, suggesting that TOR may reduce CVD risk. Results of primary coronary prevention trials estimated that a 1% increase in HDL-C was related to a 3% reduction in CVD events, and each 1 mg/dl HDL-C elevation was associated with a 3–4% decrease in cardiovascular mortality [41]. Compared with the upper limit of normal, if HDL-C was increased by 0.07 mmol/L in the TOR group (1.62 mmol/L versus 1.55 mmol/L), it would further reduce CVD risk events by 13.5% and cardiovascular mortality by 2.1–2.8%. Cholesterol-lowering medications are widely used to prevent CVD, with statins being the most commonly used drug. Although the mechanism is not yet understood, the use of statins during adjuvant endocrine therapy may prevent the recurrence of estrogen-receptor-positive early breast cancer and reduce cancer-related deaths [43, 44]. Based on the above findings, this research suggest that lipid indicators be continuously monitored during SERM treatment, that TOR is given priority in patients with cardiovascular risk factors, and that statins be used in breast cancer patients with dyslipidemia.

To the best of our knowledge, this study is the first to comprehensively evaluate the effects of TAM and TOR on lipid metabolism by combining analyses of fatty liver, fibrosis indexes and lipid levels. In addition, the joint diagnosis of fatty liver by USG and CT is innovative, which improves the sensitivity of diagnosis. In contrast to previous studies, premenopausal subjects accounted for the vast majority, while those with obesity and metabolic syndrome were rare. The main limitation of the study was the insufficient power of test. Fortunately, the primary endpoint -- that TAM was more likely to cause fatty liver disease than TOR -- was statistically significant. However, the non-statistically significant results in this study are still open to discussion. It is necessary to recruit patients, extend the duration, and complete the follow-up data to meet the sample size required to increase the reliability of the trial. A meta-analysis showed that the NAFLD incidence estimate for Asia was 52.34 per 1000 person-years [44], and whether TOR influences the progression of fatty liver (67.2 per 1000 person-years) requires further study with a larger cohort.

## Conclusion

In conclusion, TAM treatment is significantly associated with more serious fatty liver disease and liver fibrosis, while TOR improves overall lipid profiles. Given the clinical impact and medical burden of NAFLD and CVD, this research suggest regular reexaminations of liver imaging and lipid levels during SERM treatment. TOR as endocrine therapy for estrogen-receptor-positive breast cancer, especially in premenopausal patients with risk factors including obesity, diabetes, high TG levels, and low HDL-C levels, may have selective benefits.

## Data Availability

The datasets used and/or analysed during the current study are available from the corresponding author on reasonable request.

## Abbreviations

SERM: Selective estrogen receptor modulator
TAM: Tamoxifen
TOR: Toremifene
NAFLD: Non-alcoholic fatty liver disease
CVD: Cardiovascular disease
USG: Ultrasonography
CT: Computed tomography
AST: Aspartate aminotransferase
ALT: Alanine aminotransferase
NFS: NAFLD fibrosis score
APRI: AST-platelet ratio index
FIB-4: Fibrosis 4 index
BMI: Body mass index
PSM: Propensity score matching
TC: Total cholesterol
TG: Triglyceride
HDL-C: High-density lipoprotein-cholesterol
LDL-C: Low-density lipoprotein-cholesterol
